# Naturally occurring antibodies against serum amyloid A reduce IL-6 release from peripheral blood mononuclear cells

**DOI:** 10.1371/journal.pone.0195346

**Published:** 2018-04-04

**Authors:** Tadeja Kuret, Katja Lakota, Polonca Mali, Saša Čučnik, Sonja Praprotnik, Matija Tomšič, Snezna Sodin-Semrl

**Affiliations:** 1 Department of Rheumatology, University Medical Centre Ljubljana, Ljubljana, Slovenia; 2 Faculty of Mathematics, Natural Science and Information Technologies, University of Primorska, Koper, Slovenia; 3 Blood Transfusion Centre of Slovenia, Ljubljana, Slovenia; 4 Faculty of Pharmacy, University of Ljubljana, Ljubljana, Slovenia; 5 Faculty of Medicine, University of Ljubljana, Ljubljana, Slovenia; Institut National de la Santeet de la Recherche Medicale (INSERM), FRANCE

## Abstract

Serum amyloid A (SAA) is a sensitive inflammatory marker rapidly increased in response to infection, injury or trauma during the acute phase. Resolution of the acute phase and SAA reduction are well documented, however the exact mechanism remains elusive. Two inducible SAA proteins, SAA1 and SAA2, with their variants could contribute to systemic inflammation. While unconjugated human variant SAA1α is already commercially available, the variants of SAA2 are not. Antibodies against SAA have been identified in apparently healthy blood donors (HBDs) in smaller, preliminary studies. So, our objective was to detect anti-SAA and anti-SAA1α autoantibodies in the sera of 300 HBDs using ELISA, characterize their specificity and avidity. Additionally, we aimed to determine the presence of anti-SAA and anti-SAA1α autoantibodies in intravenous immunoglobulin (IVIg) preparations and examine their effects on released IL-6 from SAA/SAA1α-treated peripheral blood mononuclear cells (PBMCs). Autoantibodies against SAA and SAA1α had a median (IQR) absorbance OD (A_450_) of 0.655 (0.262–1.293) and 0.493 (0.284–0.713), respectively. Both anti-SAA and anti-SAA1α exhibited heterogeneous to high avidity and reached peak levels between 41–50 years, then diminished with age in the oldest group (51–67 years). Women consistently exhibited significantly higher levels than men. Good positive correlation was observed between anti-SAA and anti-SAA1α. Both anti-SAA and anti-SAA1α were detected in IVIg, their fractions subsequently isolated, and shown to decrease IL-6 protein levels released from SAA/SAA1α-treated PBMCs. In conclusion, naturally occurring antibodies against SAA and anti-SAA1α could play a physiological role in down-regulating their antigen and proinflammatory cytokines leading to the resolution of the acute phase and could be an important therapeutic option in patients with chronic inflammatory diseases.

## Introduction

The acute phase response (APR) is an ancient, evolutionarily conserved defense system of vertebrates regulating homeostatic disturbances caused by infections, injuries, traumas, cancer and/or immunologic disorders, ultimately leading to resolution of inflammation and healing [1, 2]. Within the APR, a number of systemic and metabolic changes occur, such as fever and anorexia on one hand, and dramatically changed levels of acute phase proteins (APPs) on the other [1], serving as promising biomarkers [3]. One of the major APPs in humans is serum amyloid A (SAA), levels of which can dramatically increase 100- to 1000-fold during APR, reaching concentrations of 1000 μg/ml [4, 5]. The SAA gene family is highly conserved, which indicates an essential role throughout evolution [6] and includes four different genes, clustered on chromosome 11p15.1 [7]. The two inducible isotypes, *SAA1* and *SAA2*, collectively called acute SAA (SAA1/SAA2) share a 93% nucleotide identity yielding proteins, 104 amino acids in length [8]. They are predominantly produced by hepatocytes during APR upon stimulation by pro-inflammatory cytokines IL-1β, IL-6 and TNF-α [9]. There are three variants of SAA1, namely SAA1α (SAA 1.1), SAA1β (SAA 1.2) and SAA1γ (SAA 1.3) and two variants of SAA2, namely SAA2α (SAA 2.1) and SAA2β (SAA 2.2) [10, 11]. SAA3 is rarely expressed in humans, with limited expression observed in mammary gland epithelial cells, while SAA4 is constitutively expressed in low levels in many different cell and tissue types [11].

Studies to date have described SAA as an activator of the inflammasome [12], stimulator of production and release of cytokines/chemokines (e.g. IL-6, TNF-α and IL-8) from human neutrophils and monocytes [13–19], up-regulator of matrix metalloproteinases [20–22], as well as player in the metabolism of HDL cholesterol [23], among other properties. Recently, human recombinant (hr) SAA1α was reported to chemoattract monocytes and dendritic cells [24], as well as neutrophils [25], similarly to hrSAA. In 2013, van den Brand et al. [26] suggested that hrSAA is more effective in induction of IL-8 transcripts in human synovial fibroblasts compared to hrSAA1α and emphasized the need to include SAA1α in assays studying biological functions of the SAA protein.

Although, SAA has been shown to play an important role in host defense [11, 27], it’s persistently high concentrations (>1000 nM) could promote injury to tissues and cells during chronic inflammatory conditions, such as joint destruction in rheumatoid arthritis (RA) [21, 28], development of atherosclerosis [29, 30], tumour pathogenesis [31] and especially reactive AA amyloidosis [32]. In the latter SAA was shown to play a major pathogenic role in amyloid deposits and was identified early on, as “the factor to be down-regulated” [12, 33]. Importantly, SAA has been described as an innate regulator of granulomatous lung inflammation in sarcoidosis acting through Toll-like receptor-2 [34], as well as mediator of glucocorticoid refractory lung inflammation in chronic obstructive pulmonary disease (COPD) [35]. Multiple groups have recently emphasized that SAA is a potential therapeutic target in the treatment of diseases associated with chronic inflammation [12], such as psoriasis [36], COPD [35], kidney involvement in autoinflammatory diseases driven by AA amyloidosis [37], as well as lung cancer metastasis [38]. Thus, acute *SAA1* and *SAA2* might also be genes well-suited to the antagonistic pleiotropy theory [39], which postulates that genetic variants with harmful effects in old ages can be tolerated, or even favoured, by natural selection at early ages. Overall, there is a critical need to control persistently up-regulated SAA in chronic inflammatory diseases.

Especially important in this regard could be natural antibodies (NAbs), which developed evolutionarily alongside innate immunity and are well conserved, since appearing in jawless fish [40, 41]. NAbs are germ-line encoded products prepared for immediate and continual protective response and play many physiological roles in maintaining homeostasis in healthy individuals [42]. They participate in clearance of a) cellular debris [43, 44], b) denatured and non-functional proteins [45], c) fibrillar aggregates or misfolded proteins [46] and could be involved in the clearance of large amounts of acute phase SAA from the circulation. A recent large Danish study on over 8000 apparently healthy blood donors (HBDs) reported that NAbs against endogenous cytokines (e.g. IL-1α, IL-6, IL-10, IFNα, GM-CSF) represent a relatively common phenomenon and that predictive factors for high, potentially neutralizing autoantibody levels may vary depending on the cytokine [47]. In addition, NAbs could prevent the interaction of pathogenic autoantibodies with its cognate antigen [48], as well as modulate the half-life and transport of cytokines and prevent inflammation and/or infection [49]. Naturally occurring autoantibodies against acute phase proteins (anti-APPs) in healthy individuals have been described [50, 51] and include anti-albumin [52], anti-CRP [53, 54] and anti-factor VIII [55] autoantibodies, among others. Although two previous studies have shown the presence of anti-SAA antibodies in HBDs [56, 57], their potential function is still unclear.

The therapeutic role of NAbs [40] has been well documented with the use of intravenous immunoglobulin (IVIg) preparation, which represents pooled IgG, extracted from plasma of thousands of healthy donors and can be an excellent source of NAbs, as well as naturally occurring anti-APPs. IVIg has been clinically proven to treat difficult cases of certain inflammatory autoimmune diseases, such as Kawasaki disease and Guillain-Barre syndrome [58]. However, till now it has been unclear whether anti-SAA antibodies are present in IVIg, and whether they could exhibit neutralizing activity against their antigen or affect cytokine release from blood cells.

The purpose of our study was to detect the presence of anti-SAA and anti-SAA1α antibodies in a larger number of HBDs and evaluate their specificity and avidity. Furthermore, we aimed to determine if anti-SAA and anti-SAA1α antibodies are present in IVIg, and explore whether isolated anti-SAA and anti-SAA1α antibody fractions from IVIg could neutralize SAA/SAA1α and/or could be useful for suppressing IL-6 release from SAA/SAA1α-stimulated peripheral blood mononuclear cells (PBMCs).

## Materials and methods

### Subject samples

Blood samples from HBDs (n = 300) within an age range of 18–67 years, with no clinical symptoms of any disease, were collected from the National Blood Transfusion Centre of Slovenia. Blood was processed and centrifuged at 3000xg for 5 min. The sera samples were aliquoted and stored at -80°C, until ready for further determination of SAA protein and anti-SAA antibody levels followed by statistical analysis. The study was conducted within the National Research Program #P3-0314 (funded by the Slovenian Research Agency), with ethical approval #99/04/15 from the Slovenian National Medical Ethics Committee. All HBDs provided informed written consent for the research and all samples were fully anonymized, before we accessed them.

### SAA proteins

SAA concentrations were measured in sera samples of all subjects by immunonephelometry (BN Prospec System, Siemens, Marburg, DE) and 300 individuals with SAA concentrations below cut-off values of 6.4 μg/ml were included in the study (7% excluded).

Recombinant Apo-SAA (hrSAA) was purchased from Peprotech, Rocky Hill, NJ. It represents a consensus SAA molecule corresponding to human Apo-SAA1α, except for the presence of an N-terminal methionine, the substitution of aspartic acid for asparagine at position 60, and histidine for arginine at position 71 (the latter two substituted residues are present in Apo-SAA2β). Apo-SAA1 (hrSAA1α, Peprotech, Rocky Hill, NJ) contains the amino acid sequence of the variant SAA1α with an additional N-terminal methionine (Table 1). Both proteins were purchased as sterile filtered and lyophilized from 5mM Tris, pH 7.8 and 7.6, respectively. The protein vials were centrifuged upon arrival and reconstituted according to manufacturer’s instructions in cell culture grade sterile water to a stock concentration of 1μg/μl. Both hrSAA (#1205CY66 and #090766) and hrSAA1α (#0613212 and #0615212) had purity >98% and endotoxin levels <0.1ng/μg of protein.

**Table 1:** Amino acid alignments of hrSAA and hrSAA1α.

PROTEIN	AA SEQUENCE
**hrSAA**	RSFFSFLGE AFDGARDMWR AYSDMREANY IGSDKYFHAR GNYDAAKRGP GGVWAAEAIS **N**ARENIQRFF G**R**GAEDSLAD QAANEWGRSG KDPNHFRPAG LPEKY
**hrSAA1α**	RSFFSFLGE AFDGARDMWR AYSDMREANY IGSDKYFHAR GNYDAAKRGP GGVWAAEAIS **D**ARENIQRFF G**H**GAEDSLAD QAANEWGRSG KDPNHFRPAG LPEKY

Asparagine (N) at amino acid (AA) 60 of SAA is substituted for aspartic acid (D) in SAA1α, while arginine (R) at AA 71 in SAA is substituted for histidine (H) in SAA1α. SAA, serum amyloid A.

### ELISA

For the detection of antibodies against SAA and its variant SAA1α, an *in-house* ELISA was utilized with optimized antigen concentrations, incubation times and temperature, sample dilutions, washing buffer and absorbance measurements [56, 57]. Briefly, high binding microtiter plates (Costar 3590, Cambridge, MA, USA) were coated with hrSAA or hrSAA1α at a concentration of 4 μg/ml, dissolved in phosphate-buffered saline (PBS, pH 7.4; 50 μl/well). The plates were incubated overnight (ON) at 4°C, washed and blocked with 1% bovine serum albumin (BSA) in PBS (200 μl /well), for 1 h at room temperature (RT). All washing steps were performed using 250 μl/well PBS in presence of 0.1% Tween-20 (pH 7.4). The samples and standards were added to the wells in a dilution of 1:100 in 1% BSA/PBS+0.1% Tween-20 (50 μl/well), incubated for 1h at RT, followed by 5 washing steps. Next, goat anti-human IgG-alkaline phosphatase conjugate was added (ACSC, AL, USA), at a dilution of 1:1000 in 1% BSA/PBS+0.1% Tween-20 (50 μl/well) and incubated for 1h, RT. Washing was performed 5 times and p-nitrophenyl phosphate in DEA buffer (2 mg/ml) was added (50 μl/well) and incubated for 15–20 min. Absorbance was measured at 450 nm at multiple time points. The measurement closest to 2.4 OD of our highest standard for SAA and 1.6 OD for SAA1α was considered as final and absorbance for an individual sample was corrected by the error correction factor of the analysis (normalization to 2.4 OD or 1.6 OD). All samples were analyzed in duplicates (average CV was 4%). The results were expressed as absolute absorbance, which was calculated by subtracting the mean absorbance of blank (1% BSA/PBS+0.1% Tween-20), from the mean absorbance of the sample. In each plate, a representative sample was used as standard (the same one in all ELISAs), serially diluted (1:100; 1:200; 1:400; 1:800; 1:1600) and an additional two samples were tested, as high and low absorbance controls.

### Specificity assays

In order to determine that binding of the anti-SAA antibodies to their respective antigens are indeed specific, we performed the following four assays:

**Fluid phase inhibition** was performed with sera samples from two HBDs, diluted 1:100, incubated ON (4°C) with different concentrations of hrSAA or hrSAA1α (e.g. 0, 0.2, 2.0 and 20 μg/ml) and analyzed using *in-house* ELISA.

#### Competition assay

Two HBDs sera samples were diluted 1:300 and a mixture of 8 pooled rabbit polyclonal anti-SAA antibodies (gift of prof. E. Malle, Institute of Molecular Biology and Biochemistry, Medical University of Graz, Austria) was added in increasing concentrations (0.15, 1.5, 15, 150, 1500 μg/ml). The rabbit antibodies target synthetic SAA peptides 1 through 8, representing the following human SAA sequences: 1–17, 14–30, 27–44, 40–63, 59–72, 68–84, 70–94, 89–104 AA, respectively. Sera samples were then analyzed using *in-house* ELISA.

#### Solid phase inhibition

Two sera samples were incubated in different dilutions (1:25; 1:100; 1:400 and 1:1600) on immobilized SAA or SAA1α for 1h at RT, before transferred from one well to another in the *in-house* ELISA.

#### Antigen blocking assay

SAA or SAA1α (4 μg/ml) was incubated with the above-mentioned rabbit polyclonal anti-SAA antibodies (pooled fraction in a ratio of 1:1) in PBS for 2h at RT. After incubation, the antigen-antibody complexes were coated onto wells in an ELISA plate followed by detection with the *in-house* ELISA.

### Avidity determination

Avidity of anti-SAA and anti-SAA1α antibodies was tested in 6 randomly selected HBDs sera samples (3 male, 3 female) with the *in-house* chaotropic ELISA. Samples were diluted 1:100 in PBS containing increasing concentrations of NaCl (137 mM, 500 mM, 1M, 2M and 2.5M). As a sample blank control, 1% BSA/PBS+0.1% Tween-20, with the same NaCl concentrations was used [57].

### Antibody fraction isolation

Octagam IVIg solution for infusion (5%; 50 mg/ml) was used as a source of anti-SAA and anti-SAA1α antibodies (Octapharma, Lachen, Switzerland). IVIg preparation consists of purified, pooled, polyspecific human IgGs obtained from a large number of HBDs (minimum of 1000 and up to 100.000 donors) [59]. Octagam IVIg was derived from >3.500 HBDs with at least 95% IgG content having a subclass distribution, similar to that found in normal human serum (60% of IgG1, 31% IgG2, 7% IgG3 and 1% IgG4).

For isolation of anti-SAA and anti-SAA1α antibodies, MicroLink Protein Coupling Kit (Thermo Scientific, Waltham, MA, USA) was used and manufacturer’s instructions followed. Briefly, 80 μl (1mg/ml) of hrSAA or hrSAA1α were immobilized directly onto beaded agarose resin with 220 μl coupling buffer (0.1M sodium phosphate, 0.15M NaCl, pH 7.2), and incubated at RT for 4h. Blocking was performed with 1M Tris-HCl, 0.05% NaN_3_ (pH 7.4) and Sodium Cyanoborohydride Solution at RT for 30 min. IgG containing IVIg was used at a concentration of 200 μg in 300 μl coupling buffer and applied to the microcolumn already coupled with hrSAA or hrSAA1α proteins. After 2 hours of incubation at RT, anti-SAA or anti-SAA1α antibodies were eluted, by adding 100 μl elution buffer (pH 2.8) to each column followed by immediate neutralization with 5 μl 1M Tris (pH 9.0). Antibody concentrations in these fractions were measured spectrophotometrically at 280 nm (Nanodrop, 2000c, Thermo Scientific, Waltham, MA, USA), aliquoted and stored at 4°C until used.

### Isolation and culture of peripheral blood mononuclear cells

Venous blood was obtained from 5 healthy volunteers (age 25–40, 3 female, 2 male) and drawn into heparin-containing tubes. Whole blood was diluted 1:1 in Dulbecco’s PBS (DPBS, Lonza, Basel, CH) without Ca^++^ and Mg^++^ and overlayed with Ficoll-Paque PLUS gradient (GE Healthcare, Chicago, IL, USA) at a density of 1.077 g/ml. Following centrifugation at 400xg for 25 min at RT, cells from the interface were collected and washed twice in DPBS by centrifugation. PBMCs were seeded in 1 ml serum-free RPMI 1640 (StemCell Technologies, Vancouver, CA) at a cell density of 3x10^5^ cells/ml and stimulated with hrSAA or hrSAA1α (at a final concentration of 1.5 μg/ml). PBMCs incubated in culture medium only, served as background control. Different concentrations of isolated anti-SAA antibodies (1.5, 3.0, 4.5, 9.0 μg/ml), anti-SAA1α antibodies (1.5, 3.0, 4.5 μg/ml), IVIg at IgG concentrations of 12, 25, 50, 100, 200, 1000, 5000 and 10.000 μg/ml, as well as anti-SAA- or anti-SAA1α-depleted IVIg (50 μg/ml) were all preincubated with hrSAA or hrSAA1α for 30 min at 37°C and then added to the PBMC suspension. After 5 hours of incubation at 37°C in a 5% CO_2_ incubator, supernatants were harvested and stored at -20°C, until tested.

### IL-6 ELISA

Released IL-6 levels from PBMCs were measured by human IL-6 ELISA (Invitrogen, Gent, BE), following manufacturer’s instructions. Briefly, supernatants from treated PBMCs were diluted 1:5 in standard diluent buffer. Biotin-labeled conjugate was incubated with supernatants for 2h. After four washes, a further incubation with streptavidin-horseradish peroxidase was performed, followed by addition of tetramethylbenzidine as substrate and stopping solution. Measurements were carried out at 450 nm with Infinite F200 Pro microplate absorbance reader (Tecan, Grödig, AT).

### Statistical analysis

The normality of distribution of anti-SAA and anti-SAA1α levels was determined by Kolmogorov-Smirnov test. Due to non-normal distribution of the data, summary statistics were expressed as medians and interquartile ranges (IQR), and nonparametric tests were performed. Mann-Whitney U test was used to compare anti-SAA and anti-SAA1α levels between males and females. Kruskal Wallis test was used for comparison of anti-SAA and anti-SAA1α levels among age groups. Spearman’s rank correlation was calculated to measure the correlation between anti-SAA and anti-SAA1α levels and between SAA concentration and anti-SAA/SAA1α antibody levels. Student t-test was used to compare mean levels of IL-6 released from differentially stimulated PBMCs in Graph Pad Prism software 5.03 (Inc., La Jolla, CA, USA). The mutual effects of gender and age on levels of anti-SAA and anti-SAA1α were evaluated using ANCOVA in SPSS statistical software package version 22.0 (Inc, Chicago, IL. USA). P values of <0.05 were regarded as statistically significant.

## Results

### Characteristics of the healthy blood donor cohort

Out of 300 apparently HBDs, 220 (73%) were men and 80 (27%) were women. The median age (IQR) of HBDs was 43.47 years (35.11–51.36), for male blood donors 45.34 years (37.06–52.73) and female blood donors 41.06 years (32.45–45.72). Men were significantly older than women (p<0.001). On the basis of their age, participants were divided into 4 groups: 18–30 years (29 men, 16 women); 31–40 years (48 men, 24 women); 41–50 years (75 men, 29 women); 51–67 years (68 men, 11 women) (Table 2).

**Table 2:** Demographics of healthy blood donors.

	MEN		WOMEN		TOTAL	
AGE GROUPS	N	%	N	%	N	%
**18–30**	29	13	16	20	45	15
**31–40**	48	22	24	30	72	24
**41–50**	75	34	29	36	104	35
**51–67**	68	31	11	14	79	26
**TOTAL**	**220**	**73**	**80**	**27**	**300**	

Age and gender distribution determined in HBDs. 4 age groups are shown. The numbers (N) and percentages (%) of men and women in each age group are indicated. HBDs, healthy blood donors.

### Levels of anti-SAA and anti-SAA1α antibodies in the sera of healthy blood donors

**Fig 1:**
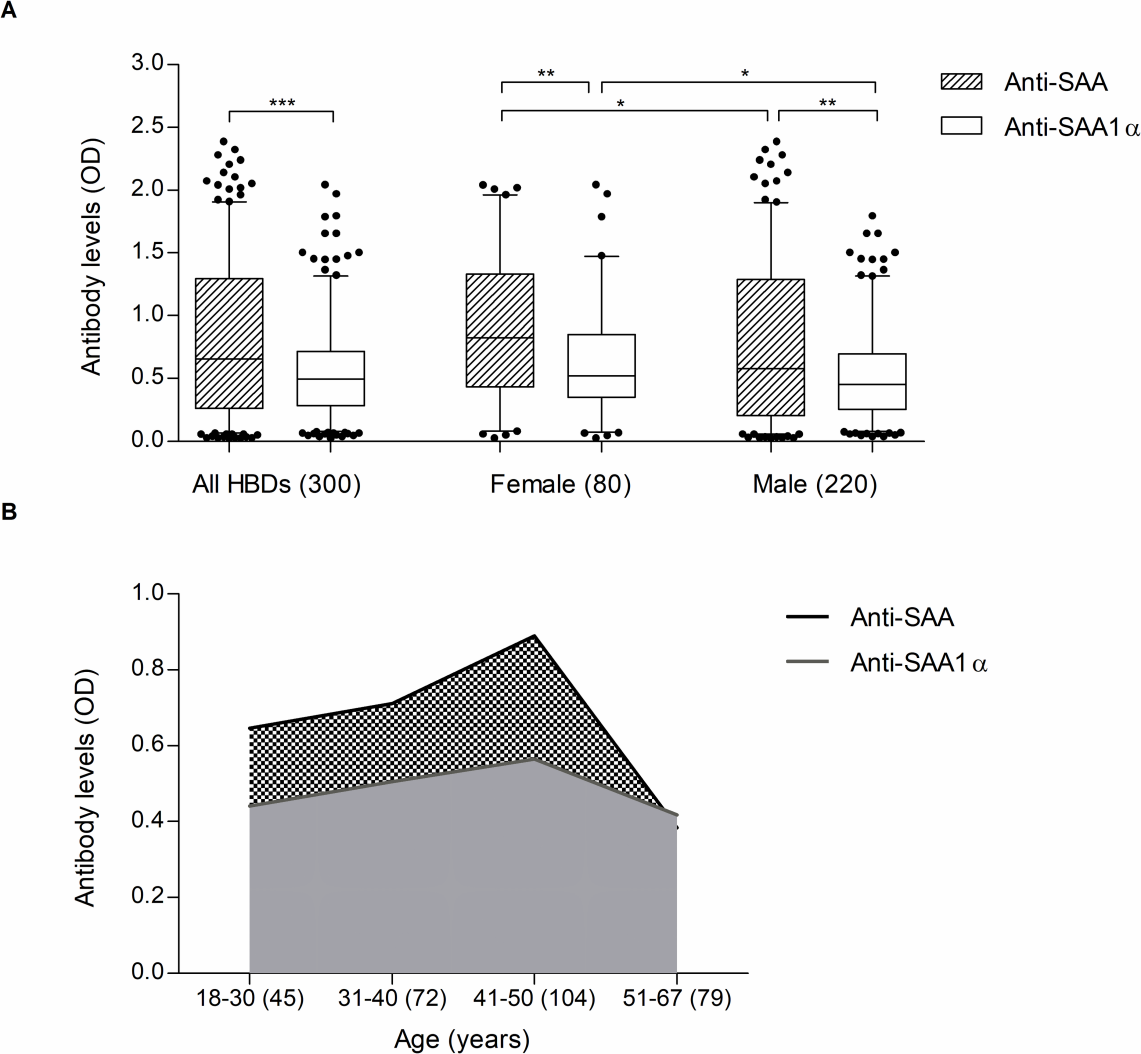
Levels of anti-SAA and anti-SAA1α antibodies. **(A)** Boxplots show the median OD (A_450_) and IQR for anti-SAA and anti-SAA1α levels in the sera of 300 HBDs (220 male and 80 female). The number of samples in each group is indicated in brackets. Whiskers represent 5^th^ and 95^th^ percentile. Medians between groups were compared using Man Whitney U-test. *p <0.05, ** p <0.01 and *** p <0.001. **(B)** Shown are medians for anti-SAA and anti-SAA1α levels in HBDs sera based on age distribution (4 groups). The number of samples in each group is indicated in brackets. HBDs, healthy blood donors; SAA, serum amyloid A.

The median (IQR) absorbance (A_450_) OD in 300 HBDs was 0.655 (0.262–1.293) for anti-SAA and significantly lower 0.493 (0.284–0.713) for anti-SAA1α (p<0.0001). Women had significantly higher levels of anti-SAA (0.823; 0.435–1.331) and anti-SAA1α (0.521; 0.348–0.851), as compared to men (0.579; 0.206–1.288; p = 0.031 for anti-SAA and 0.455; 0.254–0.696; p = 0.022 for anti-SAA1α) (Fig 1A). However, after adjustment by ANCOVA for age, the difference between women and men remained significant for anti-SAA1α (p = 0.025) but not for anti-SAA (p = 0.169). When comparing different age groups (Fig 1B), we observed lower levels in younger individuals (median;IQR) (18–30 years; 0.646; 0.196–1.218 for anti-SAA; 0.441; 0.181–0.642 for anti-SAA1α), increasing between 31 and 40 years (0.711; 0.374–1.363 for anti-SAA and 0.506; 0.348–0.772 for anti-SAA1α), reaching a peak in the middle-aged group (41–50 years; 0.890; 0.308–1.395 for anti-SAA and 0.565; 0.317–0.823 for anti-SAA1α), followed by a steep decline in median absorbance in the oldest group of individuals between 51 and 67 years (0.381; 0.179–0.785 for anti-SAA and 0.418; 0.245–0.666 for anti-SAA1α). The differences were significant for anti-SAA levels between 31–40 years and 51–67 (p<0.01) and between 41–50 and 51–67 years (p<0.001). However, there was no correlation between age and levels of anti-SAA or anti-SAA1α and no significant differences between males and females within specific age groups.

The median (IQR) concentration of SAA in the sera of all HBDs was 2.80 (2.0–4.0) μg/ml and there was no correlation observed between concentration of SAA and levels of anti-SAA/SAA1α autoantibodies (S1 Fig).

**Fig 2:**
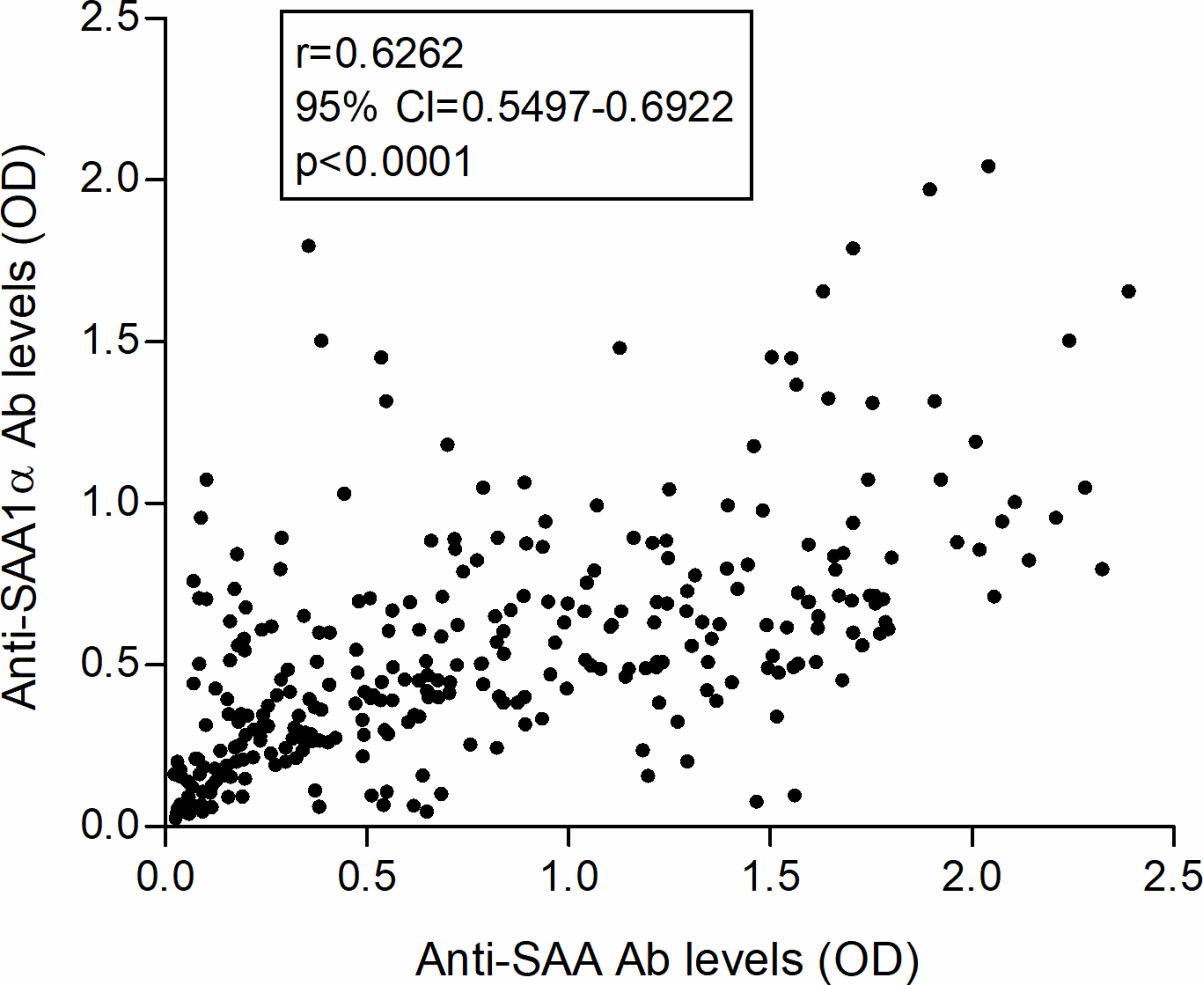
Positive correlation between levels of anti-SAA and anti-SAA1α antibodies. Correlation between levels of anti-SAA and anti-SAA1α antibodies in sera of 300 HBDs is shown. Spearman coefficient (r), 95% confidence interval (CI) and p value are indicated. Ab, antibody; HBDs, healthy blood donors; SAA, serum amyloid A.

However, there was good positive overall correlation observed between the levels of anti-SAA and anti-SAA1α IgG antibodies (Spearman coefficient r = 0.6261; 95% confidence interval: 0.5497–0.6922; p<0.0001) (Fig 2).

### Specificity of anti-SAA and anti-SAA1α ELISA

In order to verify the specificity of the detected antibodies fluid phase, competition, solid phase and antigen blocking assays were performed, which resulted in inhibition of absorbance (OD), as shown in Table 3. In the fluid phase assay the percentage of inhibition increased with elevated concentrations of hrSAA or hrSAA1α preincubated with sera samples from two HBDs. When using the competition assay, we outcompeted the human anti-SAA and anti-SAA1α within two human sera with increasing concentration of pooled rabbit anti-SAA antibodies. In the solid phase inhibition assay, serially diluted sera samples from two HBDs were transferred from one well to another and lowered levels of autoantibodies were detected by the *in-house* anti-SAA and anti-SAA1α ELISAs. The antigen blocking assay showed that preincubation of antigen with pooled polyclonal rabbit antibodies against SAA also resulted in the lowering of OD on ELISA.

**Table 3:** Specificity assays for anti-SAA and anti-SAA1α antibodies.

**FLUID PHASE INHIBITION**					
		**Anti-SAA**		**Anti-SAA1α**	
Conc. of antigen (μg/ml)		OD of inhibited sample **(% INHIBITION)**		OD of inhibited sample **(% INHIBITION)**	
		HBD1	HBD2	HBD1	HBD2
0		1.541 **(0)**	1.961 **(0)**	0.918 **(0)**	1.525 **(0)**
0.2		0.955 **(38)**	1.284 **(35)**	0.800 **(13)**	1.345 **(12)**
2.0		0.533 **(65)**	0.701 **(64)**	0.785 **(15)**	1.056 **(31)**
20.0		0.094 **(94)**	0.078 **(96)**	0.175 **(81)**	0.323 **(79)**
**COMPETITION ASSAY**					
		**Anti-SAA**		**Anti-SAA1α**	
Conc. of rabbit anti-SAA Ab (μg/ml)		OD of inhibited sample **(% INHIBITION)**		OD of inhibited sample **(% INHIBITION)**	
		HBD3	HBD4	HBD3	HBD4
0		0.834 **(0)**	0.615 **(0)**	0.427 **(0)**	0.374 **(0)**
0.15		0.770 **(8)**	0.606 **(1)**	0.418 **(2)**	0.366 **(2)**
1.5		0.761 **(9)**	0.564 **(8)**	0.406 **(5)**	0.359 **(4)**
15		0.643 **(23)**	0.532 **(13)**	0.211 **(51)**	0.279 **(25)**
150		0.441 **(47)**	0.418 **(32)**	0.141 **(67)**	0.190 **(49)**
1500		0.094 **(89)**	0.037 **(94)**	0.046 **(89)**	0.052 **(86)**
**SOLID PHASE INHIBITION**					
		**Anti-SAA**		**Anti-SAA1α**	
Sera dilution		OD of uninhibited sample	OD of inhibited sample **(% INHIBITION)**	OD of uninhibited sample	OD of inhibited sample **(% NHIBITION)**
1:25	HBD1	2.113	1.824 **(14)**	1.613	1.090 **(32)**
	HBD2	2.359	1.986 **(16)**	2.106	1.564 **(26)**
1:100	HBD1	1.582	0.823 **(48)**	1.047	0.571 **(46)**
	HBD2	1.933	1.065 **(45)**	1.576	0.738 **(53)**
1:400	HBD1	0.761	0.195 **(74)**	0.529	0.272 **(49)**
	HBD2	0.988	0.296 **(70)**	0.711	0.282 **(60)**
1:1600	HBD1	0.239	0.047 **(80)**	0.199	0.054 **(73)**
	HBD2	0.299	0.026 **(91)**	0.224	0.068 **(70)**
**ANTIGEN BLOCKING**					
		**Anti-SAA**		**Anti-SAA1α**	
Sera dilution		OD before blocking	OD after blocking	OD before blocking	OD after blocking
1:100	HBD1	1.554	0.239	1.022	0.435
	HBD2	1.926	0.173	1.543	0.666

**Fluid phase inhibition:** two HBDs samples showing percentage (%) of inhibition after preincubation with increasing concentrations of hrSAA or hrSAA1α antigens (0.2, 2, 20 μg/ml). **Competition assay:** two HBDs samples showing inhibition after adding increasing concentrations of polyclonal rabbit antibodies against SAA (0.15, 1.5, 15, 150, 1500 μg/ml). **Solid phase inhibition:** shown after transferring two samples (diluted 1:25, 1:100, 1:400, 1:1600) one well to another with 1h incubation in between. **Antigen blocking:** OD before and after blocking hrSAA or hrSAA1α with aforementioned polyclonal rabbit anti-SAA antibodies. Percentage of inhibition was calculated by subtracting “OD after inhibition” from “OD before inhibition” and dividing this by “OD before inhibition”. Ab, antibody; HBD, healthy blood donor; SAA, serum amyloid A.

### Immunoglobulin avidity

**Fig 3:**
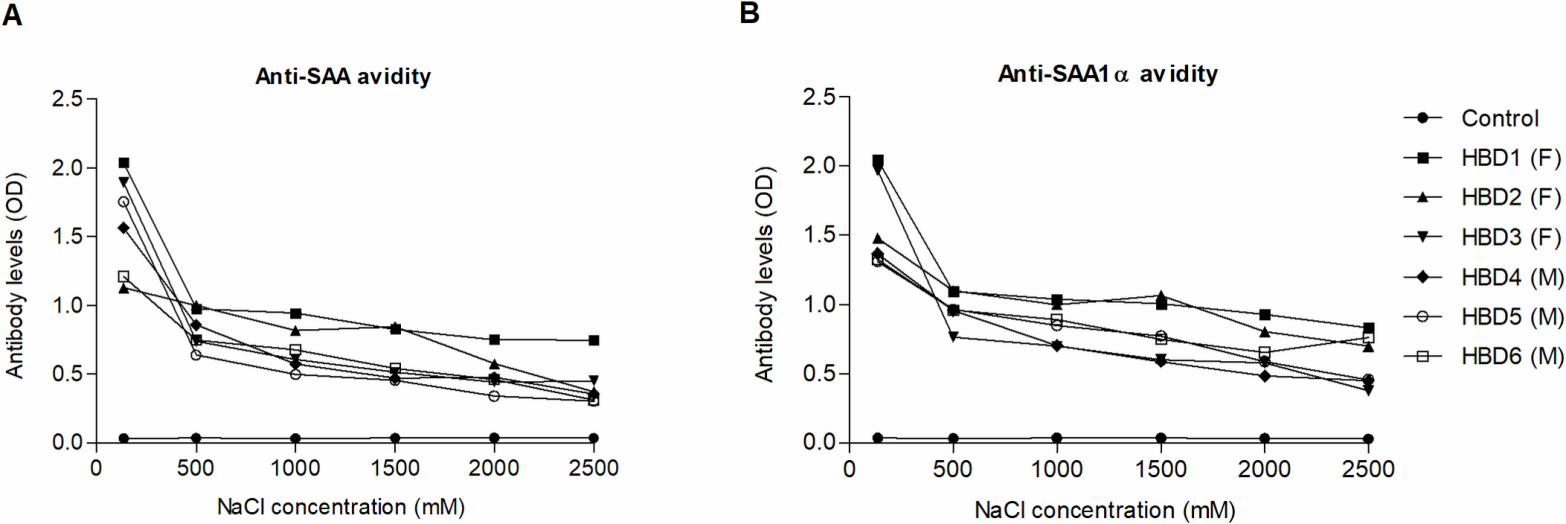
**Immunoglobulin avidity of anti-SAA (A) and anti-SAA1α (B) antibodies.** Avidity of IgG antibodies against SAA and SAA1α was determined in 6 HBDs samples (3 male, 3 female; as indicated in brackets) using increasing concentration of NaCl in sample dilution buffer. As control, 1% BSA in PBS+0.1% Tween-20 with the same NaCl concentrations, was used. BSA, bovine serum albumin; HBDs, healthy blood donors; PBS; phosphate buffered saline; SAA, serum amyloid A.

Six randomly selected sera samples (3 males, 3 females) were tested for avidity of anti-SAA and anti-SAA1α IgG antibodies. The signal rapidly decreased after increasing NaCl concentration to 500 mM and remained fairly steady upon further elevation of NaCl concentrations (Fig 3). Based on comparison between initial binding at 137 mM NaCl and binding at the higher salt concentration of 500 mM, samples can be determined to be of either low (25% or less of initial binding), heterogeneous (25–70%) or of high avidity (more than 70%) [60]. The avidity of anti-SAA antibodies was determined to be high in 1 HBD sample and heterogeneous in 5/6 samples, while the avidity of anti-SAA1α was determined as high in 4 HBD samples and heterogeneous in 2/6 HBD samples.

### Presence of anti-SAA and anti-SAA1α antibodies in intravenous immunoglobulin

**Fig 4:**
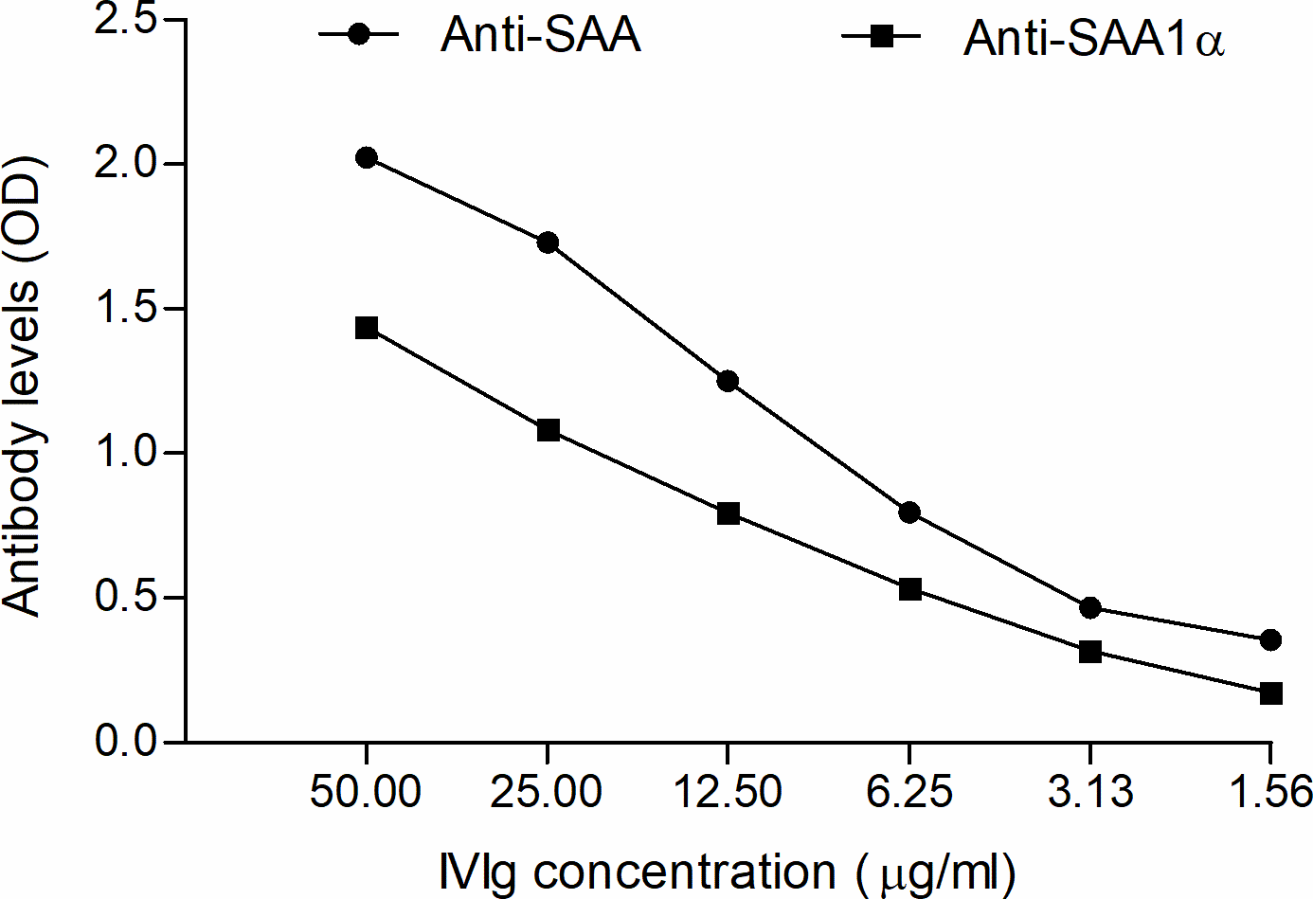
Anti-SAA and anti-SAA1α levels in IVIg. Octagam IVIg was serially diluted in sample dilution buffer (dilution range of 1.56–50 μg/ml) and analyzed for the presence of anti-SAA and anti-SAA1α antibodies using *in-house* ELISA. IVIg, intravenous immunoglobulin; SAA, serum amyloid A.

In order to investigate whether anti-SAA and anti-SAA1α antibodies were present in IVIg, an ELISA was conducted with serially diluted IVIg (IgG concentrations: 50, 25, 12.5, 6.25, 3.125 and 1.5625 μg/ml). Levels of anti-SAA and anti-SAA1α incrementally decreased with lower concentrations of IVIg. The results confirmed the presence of both anti-SAA and anti-SAA1α antibodies in IVIg, with higher levels observed for anti-SAA than anti-SAA1α antibodies (Fig 4).

### IL-6 release from hrSAA- or hrSAA1α-stimulated PBMCs

**Fig 5:**
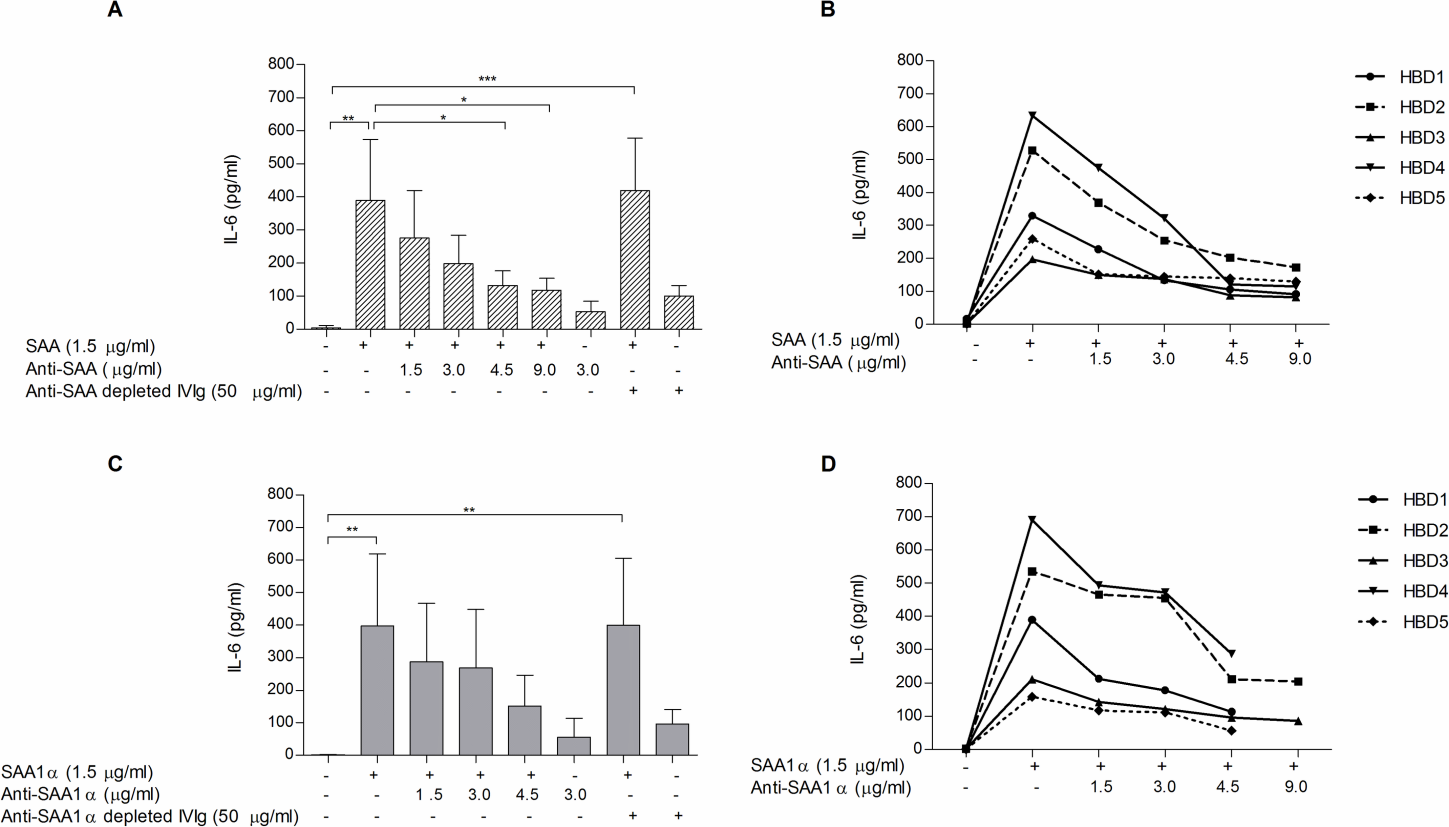
**Inhibition of IL-6 release by anti-SAA (A, B) and anti-SAA1α (C, D) isolated antibodies on hrSAA- and hrSAA1α- stimulated PBMCs.** Dose-dependent effects of isolated anti-SAA and anti-SAA1α antibodies on IL-6 release from hrSAA-treated PBMCs (**A, B**) and hrSAA1α-treated PBMCs (**C, D**). Mean±SD from five HBDs is shown for each treatment in panels A and C, while corresponding individual graphs from HBDs are shown in panels B and D. * p<0.05, ** p<0.01. PBMCs from 5 HBDs were isolated and incubated for 5h with the indicated agents. HBDs, healthy blood donors; IVIg, intravenous immunoglobulin; PBMCs, peripheral blood mononuclear cells; SAA, serum amyloid A.

Stimulation of PBMCs with hrSAA or hrSAA1α at equimolar concentrations of 125 nM (1.5 μg/ml) significantly induced IL-6 release after a 5 hour incubation, from a mean±SD basal level of 4.3±6.7 to 389.5±184.4 pg/ml with hrSAA, and 1.1±1.0 to 397.1±221.9 pg/ml with hrSAA1α stimulation (p<0.01). Levels of IL-6 gradually decreased when hrSAA or hrSAA1α were incubated with increasing doses of IVIg isolated anti-SAA (1.5, 3.0, 4.5, 9.0 μg/ml) and anti-SAA1α (1.5, 3.0, 4.5 μg/ml) antibody enriched fractions. A significant decrease in IL-6 release was observed, when adding 4.5 μg/ml (131.4±44.4 pg/ml) or 9.0 μg/ml (118.1±35.8 pg/ml) anti-SAA antibodies (p<0.05) compared to stimulation with hrSAA only (389.5±184.4 pg/ml). A similar trend was observed for anti-SAA1α antibodies, but did not reach significance. Importantly, anti-SAA- or anti-SAA1α-depleted IVIg (50 μg/ml) showed no inhibitory effects on hrSAA- or hrSAA1α-stimulated IL-6 release (Fig 5).

Interestingly, when PBMCs (isolated from 2 HBDs) were incubated with hrSAA, in presence of increasing concentrations of IVIg (up to 10 mg/ml), an effect on their IL-6 release was observed only at the highest concentration (10 mg/ml), as compared to stimulation with hrSAA alone (mean±SD 223.4±42.1 pg/ml as compared to 324.6±92.3, respectively) (S5 Table).

## Discussion

SAA is an acute phase protein with cytokine-like activities [13, 16]. All precursor SAA forms (SAA1, SAA2, SAA3 and SAA4) contain a signal peptide of 18 amino acids, which is cleaved, when the proteins are secreted (as reviewed in [11]). Of the four genes encoding SAA in humans, *SAA1* has been the most characterized [61]. In 2016, De Buck et al. emphasized that, in addition to high concentrations of SAA, also lower concentrations of SAA may be of significant relevance as diagnostic or prognostic markers for specific disease states [11].

Of the two antigens SAA and SAA1α, used as coating proteins of ELISA plates for the detection of autoantibodies in our study, SAA1α has not yet been investigated till now. In our total cohort, anti-SAA autoantibodies showed higher relative serum titers than anti-SAA1α antibodies, independent of gender or age (Fig 1). We postulate that anti-SAA antibodies include both antibodies against SAA1 and SAA2, while anti-SAA1α represent autoantibodies of a single naturally occurring antigen SAA1α in the circulation of HBDs, thus giving lower levels. Both assays were performed in order to delineate the biologically significant autoantibodies (e.g. anti-SAA1α) from the autoantibodies targeting the consensus SAA, providing characteristics of both human isoforms SAA1 and SAA2.

SAA and SAA1α sequences differ in AAs at positions 60 and 71 (Table 1). The secondary structure calculated by the computer program DSSP [62] for Protein Data Bank deposited human serum amyloid A1 (4IP8) places AA_60_ in the middle of the third of four alpha helices, while AA_71_ is on the surface of molecule, right next to a T turn on an unassigned secondary structure region. The change of the surface polar arginine for another polar AA histidine can affect electrostatic interactions, as the pI of histidine is 7.58 (side chain pKa_3_ = 6), while arginine exhibits a pI of 10.76 (pKa_3_ = 12.48), conveying their differences for ionic bond formation in physiologic pH. We suggest that this surface change could also contribute to the differential binding of the autoantibodies to their corresponding antigens observed in our assays.

There was good positive correlation between anti-SAA and anti-SAA1α levels (Fig 2). This could imply that anti-SAA1α could be used to measure biologically relevant autoantibodies in sera of healthy blood donors in the future. Interestingly, SAA antigen serum levels do not correlate to their respective anti-SAA or anti-SAA1α autoantibodies (S1 Fig), similarly to our previous study on a smaller number of HBDs [57], as well as a report on CRP and anti-CRP autoantibodies in a population of connective tissue and autoimmune diseases [63].

We observed a trend of decreased levels of anti-SAA autoantibodies after the age of 51 (Fig 1B). This goes in line with the fact that aging is known to have an effect on the immune system, leading to an impairment of both humoral and cell-mediated immunity, causing a generalized decrease in immune responsiveness. As a consequence, the duration of humoral response is shorter and the quality of produced immunoglobulins is impaired in the aged compared to younger adults [64, 65].

In Fig 3, it can be observed that the avidity is higher for sera containing lower levels of antibodies. This could be explained by the fact that high avidity antibodies form immune complexes more efficiently and are probably bound in immune complexes with their antigens in the sera. Therefore, there are less free antibodies in the sera, reaching lower levels and showing lower signals on ELISA. On the contrary, low avidity antibodies form immune complexes less effectively and therefore reach higher levels as free molecules showing higher signal on ELISA. A recent study reported on a statistically significant inverse relationship between avidity and levels of antibodies against neurofilament heavy chain in healthy blood donors, as well as patients with Alzheimer's disease [66]. Moreover, Kurtenkov et al. found negative correlation between avidity and levels of natural IgG antibodies to tumor-associated Thomsen-Friedenreich antigen, in a bound form (a phenomenon called »hidden antigens«) in healthy blood donors [67].

Although SAA and SAA1 were reported to show differences in NF-κB activation and IL-8 mRNA expression in murine NIH3T3 and human fibroblasts [26], in our assay however, we observe a similar effect of SAA, as well as SAA1α and their corresponding autoantibodies on released IL-6 from donor PBMCs (Fig 5). This related effect strengthens the fact that naturally occurring autoantibodies against SAA and SAA1α could indeed play a protective (cytokine-neutralizing) role in inflammation, as was suggested for other anti-cytokine antibodies, such as anti-IL-1α, anti-IFN-α and anti-IL-6 [47]. However, it cannot be excluded that anti-SAA and anti-SAA1α autoantibodies have other physiologically relevant biological roles, as well.

Lately, a few interesting mechanisms of how SAA is acting during pathogenicity have been presented. Ather et al. in 2013 [68] proposed that SAA stimulated antigen-presenting (dendritic) cells to promote a pro-inflammatory environment resistant to apoptosis, and therefore, resistant to the resolution of the inflammatory state. This, in turn, drove the production of T_H_17 cytokines from CD4^+^ T cells in response to antigen. This response was insensitive to corticosteroids both *in vitro* and *in vivo* [68]. Moreover, Su et al. [69] reported on glucocorticoid-stimulated production of SAA under infectious and sterile inflammation. So, SAA may actively participate in the pathogenicity of glucocorticoid-resistant lung disease [68] and therapy is actively sought to down-regulate SAA and its effects in chronic inflammatory diseases. In the last decade, tocilizumab (an anti-IL-6 biological) has been reported to successfully suppress SAA levels in polyarteritis nodosa [70] and RA [71] and was proven successful in regulating AA amyloidosis itself, even causing regression of amyloid plaques [72]. Tocilizumab was also used to treat amyloidosis secondary to different rheumatic inflammatory diseases by down-regulating IL-6 and therefore reducing sera levels of SAA [71, 73, 74]. De Buck et al. [11] stated that it made sense to block either SAA production or its activity to treat excessive inflammation and this could be achieved by reducing the production of SAA inducers, such as IL-6.

Since persistently elevated concentrations of local and systemic SAA could have harmful effects on cells and tissues, a better understanding of the players involved in SAA resolution could contribute to developing novel strategies and therapies for chronic inflammatory and autoimmune diseases [75–77], as well as AA amyloidosis [78]. In accord with this, the use of anti-SAA NAbs, isolated from IVIg, could accomplish a similar affect by controlling the ability of SAA to further induce IL-6 and other pro-inflammatory mediators.

Although the use of IVIg is considered efficient and relatively safe, there is an issue with IVIg resistance reported, especially in certain patients with Kawasaki disease [79, 80]. A number of autoantibodies against soluble and membrane-associated self-molecules involved in immune regulation are found in IVIg, such as antibodies against IL-1α, IL-6, IL-10, IFN-α and GM-CSF [47], HLA class I [81] and B-cell activating factor of the TNF family [82], to name just a few. While beneficial immunomodulatory effects of IVIg on PBMCs have been reported [83, 84], our group is the first (to our knowledge) to describe the presence of anti-SAA and anti-SAA1α antibodies in IVIg.

IVIg itself, after depletion of anti-SAA autoantibodies, slightly increased release of IL-6 from PBMCs as compared to background (Fig 5). The exact immunoregulatory mechanism of how IVIg can act has been elaborated on, however is still not entirely known or clear. Interactions with Fc-receptors have been described as an important mechanism of action of IVIg. The types of cells studied can also have influence on the potential mechanism by which cytokine production and release is altered, as different cell types (e.g. monocytes, lymphocytes) will express different amounts of Fc receptors [84]. *In vivo*, IVIg infusion can induce IL-6 and IFN-γ in plasma of patients with secondary generalized epilepsy [85]. This effect of IVIg has also been shown for patients with hypogammaglobulinaemia, where an elevation in IL-6, IL-8 and TNF-α was observed after IVIg administration [86]. In a more recent study, Wu et al. showed that incubating PBMCs from pediatric patients with IVIg alone elicited slightly higher levels of IL-6, as compared to background [83], similarly to our results.

The current study reports that IgG autoantibodies of heterogeneous to high avidity, targeting SAA and SAA1α are identified in sera of HBDs (Fig 3), confirming previous results of Lakota et al. [57] and exposing novel findings (e.g. anti-SAA1α). Females were found to have significantly higher anti-SAA and anti-SAA1α antibody levels compared to males (Fig 1A), which is in accord with the study performed by Nagele et el. [44] indicating higher prevalence of IgG autoantibodies against different proteins present on the microarray in females. In order to determine that anti-SAA autoantibodies are indeed specific and eliminate any potential false positive results, we considered the following: a) non-specific binding of secondary antibody to plastic or antigen (a sample blank control (without sera) was analyzed in antigen-coated wells, which resulted in low absorbance (OD) and was subtracted from mean OD of sera samples, b) protein-protein interactions between nonspecific IgG in the sample and the antigen (we conducted several specificity assays to address this issue, namely fluid and solid phase inhibition, competition assay and blocking of antigen with specific polyclonal antibodies raised in rabbits, as shown in Table 3), c) recognition of blocking agents by autoantibodies against dietary proteins [87], such as BSA (we tested BSA-coated wells (S1 Table), while using BSA as blocking agent, diluted sera samples with BSA-containing buffer (neutralizing serum antibody with BSA) and preincubated sera samples with BSA, human serum albumin and an irrelevant APP), d) loss of native 3D structure by binding to plastic surface and thereby exposure of neoepitopes (we addressed this by fluid phase inhibition, with IgG from sera bound to SAA antigen in fluid phase, which resulted in increased percentage of inhibition after preincubation with increasing concentrations of hrSAA), and finally e) presence of rheumatoid factor (RF) interfering in our results (RF was present in only 1/86 tested HBD). Furthermore, it needs to be pointed out that both SAA and SAA1α are human recombinant proteins, reconstituted in water, without presence of a carrier (e.g. BSA) and, as such, also do not have post-translational modifications, such as carbohydrate moieties that could potentially bind interfering antibodies targeting carbohydrates. Additionally, the isolated anti-SAA and anti-SAA1α antibodies were predominantly of the IgG isotype with heterogeneous to high avidity in HBDs, all pointing to the fact that they are specific naturally occurring antibodies.

We report that anti-SAA antibodies could be isolated from IVIg as an active antibody fraction and used to counteract SAA and down-regulate IL-6 release from SAA-stimulated PBMC in a dose-dependent manner. Interestingly, when IVIg was used to down-regulate increased IL-6 levels in SAA-treated PBMCs, it did so only in concentrations in access of 10 mg/ml, while isolated anti-SAA autoantibodies were shown to function more optimally than IVIg in suppressing IL-6 release from PBMCs already at a concentration of 4.5 μg/ml, presumably due to their exposed paratopes (Fig 5).

Taken together, this represents a novel endogenous mechanism that could regulate SAA, as well as SAA-induced cytokines, thereby limiting not only the acute phase, but also targeting chronic inflammation. We postulate that anti-SAA antibodies could be ready and available to function as natural regulators of SAA and circulatory cytokines, such as IL-6, following infections, injury and trauma [57], as well as are present in HBDs, without evidence of an APR. In the future, more studies would be necessary to better characterize these naturally occurring anti-SAA and anti-SAA1α autoantibodies isolated from IVIg for other potential functions, such as binding to their endogenous circulatory antigen and looking at effects on different cellular types, such as monocytes, lymphocytes, dendritic cells, neutrophils of HBDs, as well as patients with different chronic inflammatory diseases.

## Supporting information

S1 Fig **No correlation between sera concentration of SAA and anti-SAA (A) or anti-SAA1α (B) antibody levels.** Spearman coefficient (r), 95% confidence interval (CI) and p value are indicated. Ab, antibody; SAA, serum amyloid A.
(PDF)

S1 Table Demographics of healthy blood donors with sera concentrations of SAA and levels of anti-SAA, anti-SAA1α, and anti-BSA antibodies. Sera concentrations of SAA were determined by immunonephelometry and anti-SAA, anti-SAA1α, anti-BSA antibody levels were determined by the *in-house* ELISA. BSA, bovine serum albumin; HBD, healthy blood donor; SAA, serum amyloid A.
(PDF)

S2 Table Avidity of anti-SAA and anti-SAA1α antibodies. Avidity of IgG antibodies against SAA and SAA1α was determined in 6 HBD samples (3 male, 3 female) using increasing concentration of NaCl in sample dilution buffer. As control, 1% BSA in PBS+0.1% Tween-20 with the same NaCl concentrations, was used. BSA, bovine serum albumin; HBDs, healthy blood donors; PBS, phosphate buffered saline; SAA, serum amyloid A.
(PDF)

S3 Table Anti-SAA and anti-SAA1α antibody levels in IVIg, isolated anti-SAA and anti-SAA1α enriched fractions and anti-SAA/SAA1α depleted IVIg. Octagam IVIg and isolated anti-SAA and anti-SAA1α enriched fractions were serially diluted in sample dilution buffer and analyzed for the presence of anti-SAA and anti-SAA1α antibodies using *in-house* ELISA. IVIg, intravenous immunoglobulin; SAA, serum amyloid A.
(PDF)

S4 Table Inhibition of IL-6 release by anti-SAA and anti-SAA1α isolated antibodies on hrSAA- and hrSAA1α- stimulated PBMCs. IL-6 concentration is shown for each treatment on PBMCs, isolated from 5 different HBDs. HBDs, healthy blood donors; IVIg, intravenous immunoglobulin; PBMCs, peripheral blood mononuclear cells; SAA, serum amyloid A.
(PDF)

S5 Table IL-6 release by SAA-stimulated PBMCs in the presence of IVIg. IL-6 concentration is shown for each treatment on PBMCs, isolated from 2 different HBDs. HBDs, healthy blood donors; IVIg, intravenous immunoglobulin; PBMCs, peripheral blood mononuclear cells; SAA, serum amyloid A.
(PDF)
