# Supplementary material for: Naturally occurring antibodies against serum amyloid A reduce IL-6 release from peripheral blood mononuclear cells
Source: PLoS One. 2018 Apr 4;13(4):e0195346. doi: 10.1371/journal.pone.0195346 (PMC5884545; doi:10.1371/journal.pone.0195346)
Supplement: S1 Fig — No correlation between sera concentration of SAA and anti-SAA (A) or anti-SAA1α (B) antibody levels. Spearman coefficient (r), 95% confidence interval (CI) and p value are indicated. Ab, antibody; SAA, serum amyloid A. (PDF) [file pone.0195346.s001.pdf]

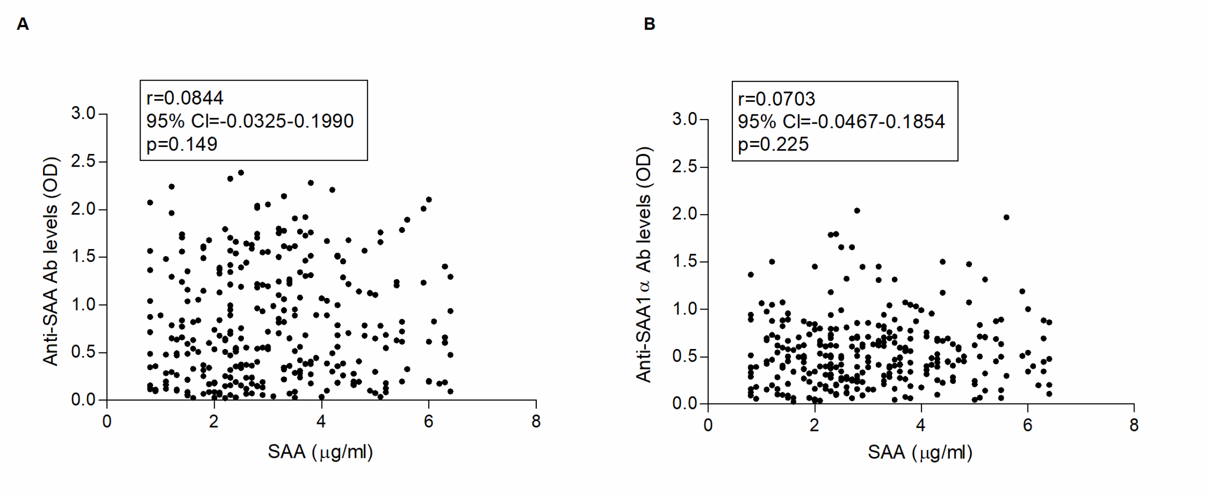

S1 Fig. No correlation between sera concentration of SAA and anti-SAA (A) or anti-SAA1α (B) antibody levels. Spearman coefficient (r), 95% confidence interval (CI) and p value are indicated. Ab, antibody; SAA, serum amyloid A.
