# Supplementary material for: Naturally occurring antibodies against serum amyloid A reduce IL-6 release from peripheral blood mononuclear cells
Source: PLoS One. 2018 Apr 4;13(4):e0195346. doi: 10.1371/journal.pone.0195346 (PMC5884545; doi:10.1371/journal.pone.0195346)
Supplement: S1 Table — Sera concentrations of SAA were determined by immunonephelometry and anti-SAA, anti-SAA1α, anti-BSA antibody levels were determined by the in-house ELISA. BSA, bovine serum albumin; HBD, healthy blood donor; SAA, serum amyloid A. (PDF) [file pone.0195346.s002.pdf]

S1 Table. Demographics of healthy blood donors with sera concentrations of SAA and levels of anti-SAA, anti-SAA1α, and anti-BSA antibodies.

HBD Number	Gender	Age (years)	SAA concentration (µg/ml)	Anti-SAA levels (OD)	Anti-SAA1α levels (OD)	Anti-BSA levels (OD)
1	F	24.08	2.80	2.040	2.044	0.052
2	F	36.35	5.10	1.761	0.713	0.043
3	F	34.79	4.10	0.101	0.315	0.058
4	F	36.26	1.60	0.332	0.343	0.051
5	F	20.25	2.20	0.508	0.397	0.070
6	F	54.68	4.30	1.506	0.528	0.045
7	F	63.01	4.90	1.127	1.480	0.047
8	F	35.44	1.20	0.651	0.469	0.041
9	F	44.27	1.00	0.890	1.065	0.041
10	F	43.53	1.20	1.963	0.880	0.039
11	F	58.12	4.75	0.785	0.505	0.032
12	F	32.36	1.40	1.243	0.885	0.045
13	F	50.66	5.60	1.896	1.970	0.045
14	F	28.12	3.40	1.250	1.044	0.024
15	M	63.15	2.49	0.649	0.418	0.046
16	M	45.31	1.19	1.293	0.729	0.069
17	M	62.16	2.81	1.703	0.700	0.051
18	F	45.72	3.18	0.703	0.413	0.001
19	M	43.48	2.34	0.724	0.623	0.105
20	M	38.56	1.45	0.217	0.213	0.049
21	M	41.16	1.76	1.595	0.873	0.044
22	M	41.66	3.20	0.817	0.651	0.055
23	M	60.00	2.40	0.534	0.390	0.038
24	F	44.39	3.19	1.246	0.832	0.045
25	M	50.90	0.77	1.565	1.367	0.043
26	M	65.74	4.66	0.408	0.600	
27	M	43.79	1.86	1.682	0.846	
28	M	49.73	3.22	1.753	1.311	
29	M	49.44	4.97	0.298	0.243	
30	M	36.48	3.71	1.305	0.559	
31	M	47.39	2.71	0.177	0.248	
32	M	50.79	0.77	0.717	0.890	
33	F	41.83	2.10	1.374	0.626	
34	F	56.38	2.81	0.147	0.158	
35	M	46.99	2.12	0.840	0.535	
36	M	29.06	2.85	1.212	0.631	
37	M	49.49	1.56	0.821	0.571	
38	M	62.09	1.48	1.039	0.666	
39	M	29.57	1.84	0.316	0.272	
40	F	54.74	2.20	0.152	0.188	

41	M	55.60	5.52	0.723	0.500
42	M	61.31	0.77	0.347	0.290
43	M	28.98	2.40	1.541	0.615
44	F	43.45	6.38	0.936	0.865
45	M	40.81	4.15	0.304	0.486
46	M	66.19	1.12	0.164	0.153
47	F	28.12	3.60	1.079	0.488
48	F	43.96	2.80	2.018	0.856
49	F	53.76	3.40	0.362	0.286
50	F	34.26	5.10	1.658	0.836
51	F	24.58	2.20	0.646	0.512
52	F	34.50	6.30	0.607	0.694
53	F	43.52	3.00	0.719	0.858
54	F	35.59	4.10	0.387	0.361
55	F	45.37	5.40	1.207	0.877
56	F	32.13	5.60	0.325	0.299
57	M	45.06	4.03	0.037	0.176
58	M	40.73	3.25	0.821	0.243
59	F	32.72	5.90	2.009	1.191
60	M	57.45	2.36	0.356	1.797
61	M	56.62	5.50	0.198	0.148
62	M	55.41	2.04	0.029	0.052
63	M	61.91	4.34	0.564	0.668
64	M	38.68	1.78	0.070	0.442
65	M	35.76	1.38	1.561	0.096
66	M	34.61	0.77	1.366	0.388
67	M	44.09	0.77	0.157	0.090
68	M	51.38	2.32	0.320	0.306
69	M	54.92	2.68	1.593	0.693
70	M	53.34	3.21	1.801	0.832
71	M	59.60	2.81	0.544	0.299
72	M	45.31	1.45	0.084	0.503
73	M	33.36	2.36	0.512	0.097
74	M	25.11	1.82	0.255	0.373
75	M	35.66	2.79	1.185	0.236
76	M	53.95	2.42	1.662	0.793
77	M	39.60	2.60	1.643	1.324
78	M	56.47	1.06	0.480	0.697
79	M	41.07	1.06	1.481	0.979
80	M	27.86	2.23	1.793	0.611
81	M	34.53	2.28	0.160	0.513
82	M	26.32	2.55	0.235	0.266
83	M	49.52	2.28	1.217	0.493
84	M	34.13	1.80	1.490	0.624
85	M	51.69	4.94	0.102	1.074

86	M	49.45	3.00	1.196	0.157
87	M	40.00	4.57	0.161	0.634
88	M	45.14	3.43	0.071	0.759
89	M	34.75	3.72	1.466	0.077
90	M	57.55	2.00	0.178	0.843
91	M	52.86	5.47	0.616	0.066
92	M	28.13	1.14	0.200	0.678
93	M	34.13	1.25	0.473	0.547
94	M	41.18	2.49	1.395	0.993
95	M	59.23	3.55	1.772	0.597
96	M	39.59	3.80	1.313	0.777
97	M	25.83	3.34	0.343	0.653
98	M	38.61	2.32	0.995	0.427
99	M	49.71	1.53	0.489	0.217
100	M	34.78	3.06	0.040	0.152
101	M	41.79	4.39	0.262	0.226
102	M	62.48	3.26	2.142	0.825
103	M	45.38	2.29	1.569	0.724
104	M	50.68	4.00	1.070	0.994
105	M	52.72	2.30	0.699	1.181
106	M	41.10	1.45	1.160	0.894
107	M	49.47	6.39	0.477	0.476
108	M	33.30	2.60	1.443	0.810
109	M	57.55	1.92	0.340	0.236
110	M	41.10	1.86	0.172	0.736
111	M	62.04	0.90	0.095	0.185
112	M	43.34	5.99	2.106	1.005
113	M	38.92	5.50	0.825	0.893
114	M	43.00	1.78	1.149	0.488
115	M	51.28	3.07	0.989	0.631
116	M	37.26	1.39	0.838	0.384
117	M	41.73	2.86	0.553	0.606
118	M	34.65	4.25	0.186	0.254
119	M	32.33	2.86	0.934	0.333
120	M	55.05	4.70	1.141	0.463
121	M	42.15	3.83	0.274	0.192
122	M	29.21	6.23	0.176	0.201
123	M	48.72	4.37	1.459	1.177
124	M	47.26	6.05	0.827	0.402
125	M	43.45	1.71	1.056	0.499
126	M	39.19	3.01	1.558	0.493
127	M	58.20	0.89	0.358	0.393
128	M	52.56	4.56	0.173	0.245
129	M	27.56	2.73	0.364	0.265
130	M	55.02	3.33	1.617	0.614

131	M	42.64	2.82	0.404	0.260
132	M	28.07	4.31	0.684	0.102
133	M	53.29	2.95	0.537	0.448
134	M	33.10	2.96	0.563	0.391
135	M	50.63	3.47	0.889	0.713
136	M	54.16	2.59	0.372	0.113
137	M	48.30	5.40	1.244	0.690
138	M	35.36	2.92	1.552	1.450
139	M	45.87	3.61	0.564	0.493
140	M	35.04	2.12	0.678	0.398
141	M	22.87	3.24	1.504	1.453
142	M	51.10	5.96	0.196	0.545
143	M	51.50	2.24	0.254	0.311
144	M	54.16	1.33	0.111	0.105
145	M	52.83	1.40	1.705	0.599
146	M	30.20	1.65	0.839	0.603
147	M	48.47	3.76	0.179	0.560
148	M	49.80	5.95	0.203	0.343
149	M	25.88	3.30	0.325	0.212
150	M	53.80	4.53	1.219	0.694
151	M	54.78	4.70	0.194	0.581
152	M	34.43	2.76	0.154	0.394
153	M	41.41	0.79	2.074	0.943
154	M	30.87	2.86	0.056	0.091
155	M	29.29	3.66	1.728	0.560
156	M	60.42	4.95	1.108	0.622
157	M	56.18	5.41	0.629	0.450
158	M	39.35	3.82	0.406	0.439
159	M	41.00	5.22	0.125	0.141
160	M	43.10	2.19	0.023	0.162
161	M	53.72	1.06	0.124	0.426
162	M	46.20	2.88	0.192	0.093
163	M	46.21	1.95	0.535	1.451
164	M	52.73	2.34	0.057	0.139
165	M	32.86	3.91	0.443	1.030
166	M	44.91	1.77	1.493	0.492
167	M	48.79	2.92	0.137	0.235
168	M	37.70	5.00	0.075	0.210
169	M	60.40	0.95	0.114	0.059
170	M	37.26	1.83	0.370	0.370
171	M	48.77	1.33	0.263	0.620
172	M	23.36	5.12	0.036	0.068
173	M	60.47	2.62	0.553	0.286
174	M	63.38	2.57	0.122	0.179
175	M	51.11	6.34	0.188	0.347

176	M	38.68	2.16	0.629	0.609
177	M	53.72	5.21	0.084	0.706
178	M	33.16	4.20	2.207	0.956
179	M	25.12	0.77	0.134	0.158
180	M	51.80	1.83	1.614	0.509
181	M	45.13	2.31	0.893	0.316
182	M	53.06	1.20	0.297	0.200
183	M	54.78	2.04	0.085	0.161
184	M	27.60	2.07	0.059	0.040
185	M	23.06	3.43	1.225	0.384
186	M	45.36	2.05	1.332	0.633
187	M	43.04	3.76	1.761	0.691
188	M	29.36	2.45	2.388	1.656
189	M	48.79	3.70	1.923	1.073
190	M	39.45	1.05	0.287	0.455
191	M	46.30	0.77	0.115	0.124
192	M	53.44	5.19	0.181	0.324
193	M	60.92	3.58	0.309	0.417
194	M	49.81	3.03	2.055	0.711
195	M	49.84	4.09	0.891	0.401
196	M	49.20	4.45	1.678	0.453
197	M	50.55	3.26	1.780	0.705
198	M	26.38	6.38	0.093	0.108
199	M	52.58	1.63	0.630	0.340
200	M	42.52	1.16	2.242	1.504
201	M	46.93	3.55	0.242	0.345
202	M	30.10	2.74	0.074	0.062
203	M	57.27	1.36	0.658	0.405
204	M	35.91	2.26	2.323	0.797
205	M	34.08	1.31	0.493	0.284
206	M	56.26	2.26	0.949	0.696
207	M	39.32	3.53	1.907	1.315
208	M	34.45	5.10	0.782	0.503
209	M	50.94	4.53	0.706	0.447
210	M	49.40	2.12	1.130	0.666
211	M	52.73	3.46	0.092	0.045
212	M	40.68	2.78	1.746	0.714
213	M	44.26	2.52	0.339	0.275
214	M	54.07	2.46	0.756	0.254
215	M	55.98	1.89	0.090	0.067
216	M	31.68	3.79	2.280	1.049
217	M	58.59	2.45	0.157	0.347
218	M	56.79	2.69	0.240	0.609
219	M	43.42	1.68	0.507	0.706
220	M	49.79	1.54	0.088	0.956

221	M	50.23	1.32	0.639	0.158
222	M	37.25	2.78	1.219	0.509
223	M	52.55	2.32	0.244	0.316
224	M	33.43	2.44	0.030	0.200
225	M	22.81	3.48	0.030	0.161
226	M	18.67	1.96	0.028	0.040
227	M	47.86	0.79	0.873	0.384
228	M	39.48	5.45	1.786	0.633
229	M	28.37	4.32	0.998	0.691
230	M	52.02	2.68	1.632	1.656
231	M	49.50	1.39	1.743	1.073
232	M	37.00	1.48	0.593	0.455
233	M	57.76	2.55	0.067	0.124
234	M	41.65	3.40	1.271	0.324
235	M	36.18	4.21	0.494	0.417
236	M	57.83	5.20	0.686	0.711
237	M	30.91	3.35	0.653	0.401
238	M	46.44	4.80	0.676	0.453
239	M	39.55	1.32	0.102	0.705
240	M	42.60	2.36	0.549	0.108
241	M	20.05	3.83	1.516	0.340
242	M	53.01	4.37	0.386	1.504
243	M	47.37	6.04	0.617	0.345
244	M	24.79	3.78	0.381	0.062
245	M	52.77	4.58	0.278	0.405
246	M	46.33	3.60	0.285	0.797
247	F	41.89	4.60	0.200	0.284
248	M	44.48	3.40	1.597	0.696
249	F	29.44	5.20	0.549	1.315
250	F	43.69	4.82	1.568	0.503
251	F	58.58	6.30	1.405	0.447
252	F	42.71	4.42	1.292	0.666
253	F	51.23	5.02	0.648	0.045
254	F	49.25	4.08	1.671	0.714
255	F	34.81	3.59	0.422	0.275
256	F	41.89	2.02	0.188	0.344
257	F	42.88	1.59	0.541	0.067
258	F	31.06	1.20	0.789	1.049
259	F	46.09	3.70	0.383	0.267
260	F	40.66	3.54	0.288	0.893
261	F	24.76	2.42	0.192	0.207
262	F	46.63	3.23	1.103	0.618
263	F	49.79	2.33	0.236	0.280
264	F	37.77	6.32	0.660	0.885
265	F	23.83	3.30	0.943	0.944

266	F	34.26	3.50	1.618	0.650
267	F	57.97	2.26	1.704	1.789
268	F	42.01	4.30	1.520	0.476
269	F	38.21	3.46	0.516	0.406
270	F	31.30	1.46	1.355	0.582
271	F	48.50	2.09	0.081	0.209
272	F	35.41	3.21	0.857	0.669
273	F	24.34	1.36	0.773	0.824
274	F	56.72	2.25	0.472	0.380
275	F	37.12	4.13	1.045	0.755
276	F	41.45	1.46	0.058	0.091
277	F	42.82	2.25	1.419	0.736
278	F	28.93	0.77	1.041	0.515
279	F	22.72	2.25	1.704	0.940
280	F	29.00	4.30	0.788	0.441
281	F	42.14	1.63	0.026	0.025
282	F	41.83	3.52	0.955	0.470
283	F	45.72	1.92	0.602	0.323
284	F	27.01	2.32	1.345	0.509
285	F	35.33	1.99	0.738	0.788
286	F	22.67	2.46	0.376	0.510
287	F	20.95	1.48	0.050	0.069
288	F	32.72	3.32	1.063	0.793
289	F	42.01	3.57	1.344	0.422
290	F	53.84	3.69	0.381	0.600
291	F	28.12	2.48	1.191	0.489
292	F	35.30	5.90	1.233	0.508
293	F	46.63	3.91	0.895	0.875
294	F	46.86	2.77	0.966	0.569
295	F	36.26	3.67	0.684	0.589
296	F	37.75	0.79	0.489	0.331
297	M	40.04	3.34	0.221	0.301
298	M	47.77	2.12	1.392	0.798
299	M	58.20	4.32	0.358	0.393
300	M	44.21	6.38	1.293	0.203

Sera concentrations of SAA were determined by immunonephelometry and anti-SAA, anti-SAA1α, anti-BSA antibody levels were determined by the *in-house* ELISA. BSA, bovine serum albumin; HBD, healthy blood donor; SAA, serum amyloid A.
