# Supplementary material for: Naturally occurring antibodies against serum amyloid A reduce IL-6 release from peripheral blood mononuclear cells
Source: PLoS One. 2018 Apr 4;13(4):e0195346. doi: 10.1371/journal.pone.0195346 (PMC5884545; doi:10.1371/journal.pone.0195346)
Supplement: S2 Table — Avidity of IgG antibodies against SAA and SAA1α was determined in 6 HBD samples (3 male, 3 female) using increasing concentration of NaCl in sample dilution buffer. As control, 1% BSA in PBS+0.1% Tween-20 with the same NaCl concentrations, was used. BSA, bovine serum albumin; HBDs, healthy blood donors; PBS, phosphate buffered saline; SAA, serum amyloid A. (PDF) [file pone.0195346.s003.pdf]

S2 Table. Avidity of anti-SAA and anti-SAA1α antibodies.

		NaCl (mM)					
		**137**	**500**	**1000**	**1500**	**2000**	**2500**
**HBD Number**	**Gender**	**Anti-SAA levels (OD)**					
1	F	2.040	0.976	0.945	0.829	0.753	0.750
7	F	1.127	1.000	0.819	0.847	0.576	0.373
13	F	1.896	0.738	0.607	0.515	0.443	0.450
25	M	1.565	0.859	0.574	0.471	0.476	0.357
28	M	1.753	0.639	0.499	0.455	0.340	0.305
36	M	1.212	0.749	0.678	0.542	0.460	0.313
Control	N/A	0.034	0.035	0.034	0.035	0.037	0.035
		**Anti-SAA1α levels (OD)**					
1	F	2.044	1.095	1.039	1.006	0.929	0.832
7	F	1.480	1.099	0.998	1.066	0.804	0.699
13	F	1.970	0.765	0.698	0.601	0.579	0.377
25	M	1.367	0.958	0.704	0.588	0.484	0.450
28	M	1.311	0.964	0.846	0.772	0.588	0.456
36	M	1.327	0.959	0.893	0.748	0.653	0.762
Control	N/A	0.034	0.033	0.035	0.034	0.033	0.029

Avidity of IgG antibodies against SAA and SAA1α was determined in 6 HBD samples (3 male, 3 female) using increasing concentration of NaCl in sample dilution buffer. As control, 1% BSA in PBS+0.1 % Tween-20 with the same NaCl concentrations, was used. BSA, bovine serum albumin; HBDs, healthy blood donors; PBS, phosphate buffered saline; SAA, serum amyloid A.
