# Supplementary material for: Naturally occurring antibodies against serum amyloid A reduce IL-6 release from peripheral blood mononuclear cells
Source: PLoS One. 2018 Apr 4;13(4):e0195346. doi: 10.1371/journal.pone.0195346 (PMC5884545; doi:10.1371/journal.pone.0195346)
Supplement: S3 Table — Octagam IVIg and isolated anti-SAA and anti-SAA1α enriched fractions were serially diluted in sample dilution buffer and analyzed for the presence of anti-SAA and anti-SAA1α antibodies using in-house ELISA. IVIg, intravenous immunoglobulin; SAA, serum amyloid A. (PDF) [file pone.0195346.s004.pdf]

**S3 Table. Anti-SAA and anti-SAA1α antibody levels in IVIg, isolated anti-SAA and anti-SAA1α enriched fractions and anti-SAA/SAA1α depleted IVIg.**

| **IVIg** | | | **Isolated anti-SAA enriched fraction** | **Isolated anti-SAA1α enriched fraction** | **Anti-SAA depleted IVIg** | **Anti-SAA1α depleted IVIg** |
|---|---|---|---|---|---|---|
| Concentration (μg/ml) | Anti-SAA level (OD) | Anti-SAA1α level (OD) | Anti-SAA level (OD) | Anti-SAA1α level (OD) | Anti-SAA level (OD) | Anti-SAA1α level (OD) |
| 50 | 2.024 | 1.436 | 2.773 | 1.758 | 0.105 | 0.112 |
| 25 | 1.731 | 1.081 | 2.361 | 1.496 | | |
| 20 | 1.570 | 0.986 | 1.6440 | 1.287 | | |
| 12.5 | 1.252 | 0.796 | 1.4196 | 0.979 | | |
| 10 | 1.016 | 0.633 | 1.3840 | 0.879 | | |
| 6.25 | 0.798 | 0.534 | 1.1880 | 0.658 | | |
| 5 | 0.657 | 0.411 | 1.0450 | 0.558 | | |
| 3.13 | 0.467 | 0.319 | 0.8050 | 0.419 | | |
| 2.5 | 0.456 | 0.289 | 0.7280 | 0.384 | | |
| 1.56 | 0.354 | 0.175 | 0.5037 | 0.271 | | |
| 1.25 | 0.351 | 0.225 | 0.4160 | 0.232 | | |
| 0.63 | 0.297 | 0.193 | 0.2980 | 0.169 | | |
| 0 | 0.037 | 0.046 | 0.037 | 0.046 | 0.037 | 0.046 |

Octagam IVIg and isolated anti-SAA and anti-SAA1α enriched fractions were serially diluted in sample dilution buffer and analyzed for the presence of anti-SAA and anti-SAA1α antibodies using *in-house* ELISA. IVIg, intravenous immunoglobulin; SAA, serum amyloid A.
