# Supplementary material for: Naturally occurring antibodies against serum amyloid A reduce IL-6 release from peripheral blood mononuclear cells
Source: PLoS One. 2018 Apr 4;13(4):e0195346. doi: 10.1371/journal.pone.0195346 (PMC5884545; doi:10.1371/journal.pone.0195346)
Supplement: S4 Table — IL-6 concentration is shown for each treatment on PBMCs, isolated from 5 different HBDs. HBDs, healthy blood donors; IVIg, intravenous immunoglobulin; PBMCs, peripheral blood mononuclear cells; SAA, serum amyloid A. (PDF) [file pone.0195346.s005.pdf]

S4 Table. Inhibition of IL-6 release by anti-SAA and anti-SAA1α isolated antibodies on hrSAA- and hrSAA1α- stimulated PBMCs.

**PBMC STIMULATION**									
**SAA (μg/ml)**	0	1.5	1.5	1.5	1.5	1.5	0	1.5	0
**Anti-SAA (μg/ml)**	0	0	1.5	3	4.5	9	3	0	0
**Anti-SAA depleted IVIg (μg/ml)**	0	0	0	0	0	0	0	50	50
**IL-6 CONCENTRATION (pg/ml)**									
**1**	16.199	329.444	227.462	133.993	105.636	91.036	24.249	259.621	100.974
**2**	2.175	527.812	369.872	254.138	202.837	172.548	59.470	524.950	81.015
**3**	1.536	197.614	149.534	137.797	87.510	82.451	76.736	254.734	155.259
**4**	1.536	633.375	475.090	322.121	121.136	114.653	15.719	616.349	83.188
**5**	0.000	259.329	151.978	144.155	139.753	129.963	90.003	435.320	79.756

**PBMC STIMULATION**									
**SAA1α (μg/ml)**	0	1.5	1.5	1.5	1.5	1.5	0	1.5	0
**Anti-SAA1α (μg/ml)**	0	0	1.5	3	4.5	9	3	0	0
**Anti-SAA1α depleted IVIg (μg/ml)**	0	0	0	0	0	0	0	50	50
**IL-6 CONCENTRATION (pg/ml)**									
**1**	0.000	389.857	212.446	177.927	112.778		153.445	391.547	110.356
**2**	2.175	535.516	465.669	454.850	210.683	204.356	54.657	524.954	100.974
**3**	1.536	210.987	143.177	121.627	95.477	85.719	36.465	99.939	81.015
**4**	1.536	690.606	493.253	472.100	287.217		1.536	643.918	155.259
**5**	0.000	158.336	117.206	110.807	56.170		34.363	337.223	34.188

IL-6 concentration is shown for each treatment on PBMCs, isolated from 5 different HBDs. HBDs, healthy blood donors; IVIg, intravenous immunoglobulin; PBMCs, peripheral blood mononuclear cells; SAA, serum amyloid A.
