# Supplementary material for: Naturally occurring antibodies against serum amyloid A reduce IL-6 release from peripheral blood mononuclear cells
Source: PLoS One. 2018 Apr 4;13(4):e0195346. doi: 10.1371/journal.pone.0195346 (PMC5884545; doi:10.1371/journal.pone.0195346)
Supplement: S5 Table — IL-6 concentration is shown for each treatment on PBMCs, isolated from 2 different HBDs. HBDs, healthy blood donors; IVIg, intravenous immunoglobulin; PBMCs, peripheral blood mononuclear cells; SAA, serum amyloid A. (PDF) [file pone.0195346.s006.pdf]

**S5 Table. IL-6 release by SAA-stimulated PBMCs in the presence of IVIg.**

| **PBMC STIMULATION** | | | | | | | | | | | |
|---|---|---|---|---|---|---|---|---|---|---|---|
| **SAA (µg/ml)** | 0 | 1.5 | 1.5 | 1.5 | 1.5 | 1.5 | 0 | 1.5 | 1.5 | 1.5 | 0 |
| **IVIg (µg/ml)** | 0 | 0 | 12 | 25 | 50 | 100 | 200 | 1000 | 5000 | 10000 | 200 |
| **IL-6 CONCENTRATION (pg/ml)** | | | | | | | | | | | |
| **1** | 16.199 | 329.444 | 411.289 | 455.878 | 461.253 | 480.103 | | | | | 100.974 |
| **2** | 2.175 | 527.812 | 716.215 | 736.113 | 781.400 | 807.883 | | | | | 81.015 |
| **3** | **0.000** | **259.329** | **269.077** | **264.451** | **266.505** | **256.775** | **267.019** | **251.170** | **221.946** | **193.666** | **79.756** |
| **4** | **0.000** | **389.857** | **416.717** | **381.399** | **352.509** | **398.939** | **388.726** | **363.543** | **295.592** | **253.206** | **100.580** |

IL-6 concentration is shown for each treatment on PBMCs, isolated from 2 different HBDs. HBDs, healthy blood donors; IVIg, intravenous immunoglobulin; PBMCs, peripheral blood mononuclear cells; SAA, serum amyloid A.
